# Riding the Rhythm of Melatonin Through Pregnancy to Deliver on Time

**DOI:** 10.3389/fendo.2019.00616

**Published:** 2019-09-13

**Authors:** Ronald McCarthy, Emily S. Jungheim, Justin C. Fay, Keenan Bates, Erik D. Herzog, Sarah K. England

**Affiliations:** ^1^Department of Obstetrics and Gynecology, Washington University School of Medicine, St. Louis, MO, United States; ^2^Department of Biology, University of Rochester, Rochester, NY, United States; ^3^Department of Biology, Washington University, St. Louis, MO, United States

**Keywords:** melatonin, pregnancy, gestation, parturition, circadian, chronodisruption, fetal outcomes

## Abstract

Pregnancy is influenced by the circadian (“circa” or approximately; diēm or day) system, which coordinates physiology and behavior with predictable daily changes in the environment such as light/dark cycles. For example, most species deliver around a particular time of day. In mammals, circadian rhythms are controlled by the master circadian pacemaker, the suprachiasmatic nucleus. One key way that the suprachiasmatic nucleus coordinates circadian rhythms throughout the body is by regulating production of the sleep-promoting hormone melatonin. Serum melatonin concentration, which peaks at night and is suppressed during the day, is one of the best biological indicators of circadian timing. Circadian misalignment causes maternal disturbances in the temporal organization of many physiological processes including melatonin synthesis, and these disturbances of the circadian system have been linked to an increased risk for pregnancy complications. Here, we review evidence that melatonin helps regulate the maternal and fetal circadian systems and the timing of birth. Finally, we discuss the potential for melatonin-based therapeutic strategies to alleviate poor pregnancy outcomes such as preeclampsia and preterm birth.

## Introduction

Pregnancy requires coordination of numerous physiological systems, including metabolic, endocrine, and circadian (1). This review focuses on a key component of the circadian system, the sleep-promoting hormone melatonin. However, to appreciate the role of melatonin in pregnancy, it is essential to understand a few facts about circadian rhythms, which coordinate physiological functions with daily environmental cues (e.g., 24-h light/dark cycle, temperature, and food availability).

At a molecular level, circadian rhythms are controlled by a group of core clock genes working together in a transcriptional/translational feedback loop. In the dark phase, the transcription factors CLOCK and BMAL1 reach a high concentration (2, 3), heterodimerize, and activate expression of the Period (*PER1, PER2, PER3*) and Cryptochrome (*CRY1, CRY2*) genes. PER and CRY proteins accumulate in the cytoplasm, reach their highest concentration in the light period, dimerize, translocate to the nucleus, and interfere with BMAL1-CLOCK to prevent their own transcription. Once transcription/translation of CRY and PER are prevented and the accumulated proteins degrade, CLOCK/BMAL1 dimers are again able to bind, allowing transcription and translation of CRY and PER proteins. Additionally, the BMAL1-CLOCK dimers activate transcription of the retinoic acid-related orphan nuclear receptors *REV-ERBA* and *RoR*α, which inhibits *BMAL1* transcription. Conversely, the CRY1 protein inhibits transcription of *REV-ERBA*, allowing RoR to activate *BMAL1* transcription. These evolutionarily well-conserved positive and negative feedback loops regulate circadian rhythms in nearly every cell in the body.

In mammals, the clocks throughout the body (termed peripheral oscillators) are controlled by the master circadian pacemaker, the suprachiasmatic nucleus (SCN), which synchronizes to external light/dark cycles via signals received from the melanopsin-containing retinal ganglion cells (1, 4–6). The SCN neurons, which have a high firing rate during the light period and a low firing rate during the dark period, synchronize the peripheral oscillators via a multisynaptic pathway. The SCN projects to the paraventricular nucleus, which connects to the intermediolateral cell column of the T1-T3 segment of the spinal cord. This cell column connects to the superior cervical ganglion, which connects to the pineal gland (7). The pineal gland synthesizes and secretes melatonin (N-acetyl-5-methoxytriptamine (7). Melatonin functions with other neurotransmitters, such as vasoactive intestinal polypeptide, to synchronize circadian rhythms throughout the body.

## Melatonin Synthesis and Mechanisms of Action

Melatonin is synthesized most highly in the dark phase as a result of the following pathway. First, the precursor L-tryptophan is converted to 5-hydroxytryptophan by tryptophan hydroxylase, an enzyme whose expression is highest during the dark phase (8–10). Aromatic amino decarboxylase then converts 5-hydroxytryptophan to serotonin (5-hydroxytryptamine) (11, 12). At night, the neurotransmitter norepinephrine activates the pineal gland β-adrenergic receptors, leading to increased cellular cAMP and increased expression of serotonin N-acetyl transferase, which converts serotonin to N-acetyl-serotonin (13). N-acetyl-serotonin is then methylated by hydroxyindole-o-methyl transferase to become melatonin (14–16), which is released into the circulatory system and cerebrospinal fluid (11, 17). Although melatonin is primarily secreted by the pineal gland (18–20), the enzymes that convert serotonin to melatonin are also expressed in other tissues/cell types including the brain (21), retinal photoreceptor cells (22, 23), immune system (24–26), skin (27), gastrointestinal tract (28, 29), and reproductive tract (20). In addition to being regulated by the SCN, melatonin in rats regulates the SCN by inducing expression of *Per1* and *Per2* genes to help reset the master clock (30).

The primary functions of melatonin are to relay information to the body regarding the length of the light and dark cycles (photoperiod) and to signal the body about seasonal changes in the photoperiod. As the dark period becomes longer in winter, melatonin is secreted for a longer period of time. The daily rhythm of melatonin synthesis allows the body to respond to the changing seasons by altering many physiological functions, such as sleep duration, weight, temperature, blood pressure, and in general, control of mammalian reproduction (17, 31–34).

Melatonin functions via several mechanisms. First, it activates the G-protein-coupled receptors MTNR1A and MTNR1B, which are expressed in the SCN, brain, and numerous peripheral organs including the retina, pars tuberalis, cerebral and peripheral arteries, kidney, pancreas, adrenal cortex, testes, immune system, uterus, and placenta (35). Second, melatonin can bind members of the retinoid related orphan nuclear hormone receptor subfamily RZR/ROR, which regulate many processes including immunity, metabolic pathways, embryonic development, and circadian rhythms (36). Melatonin is thought to bind RZR/RORβ, which is primarily expressed in the retina, brain, and pineal gland, and RZR/RORα, which is highly expressed in the brain, liver, skeletal muscle, skin, lung, kidney, thymus, adipose tissue, and placenta (37–40). Third, melatonin can modulate intracellular calcium by binding calmodulin (41). Finally, melatonin and its metabolites, including cyclic-3-hydroxymelatonin, can scavenge reactive oxygen species (e.g., superoxide radical, hydroxyl radical, and hydrogen peroxide) (42–45) and thereby protect tissues from oxidative damage. Some or all of these mechanisms may explain the many roles of melatonin in reproduction, as described in the rest of this review.

## Roles of Circadian Rhythms and Melatonin in Timing Pregnancy

In many species, reproduction is limited to particular seasons, indicating that the circadian system controls some aspects of the timing of pregnancy. For example, hamsters are more fertile and have a more regular 4-day estrous cycle in summer (long light period) than in other seasons. The proestrus stage is characterized by an afternoon surge in luteinizing hormone (LH) and follicle-stimulating hormone (FSH), whereas in diestrus, LH and FSH concentrations are low or virtually absent. When female hamsters were exposed to winter-like (long dark period) conditions, they became acyclic, anovulatory, and showed a daily afternoon surge of LH and a small increase in FSH, suggesting they were in prolonged proestrus (46, 47). When the female hamsters were maintained in a long light period and injected with melatonin for several days, their estrus cycle became acyclic, and secretion of LH and FSH had a pattern similar to that found during a long dark period (48). Although it is likely that melatonin helps control seasonal reproduction in other species, few experiments have been done to test this idea.

## Roles of Circadian Regulation and Melatonin During Pregnancy: Hormones, Metabolism, Body Temperature, and Maternal Activity

During pregnancy, females undergo numerous physiological changes to support fetal development and adapt to the stresses imposed on their bodies. The circadian and melatonin systems play important roles in some of the most notable changes, which occur in the endocrine system, metabolism, core body temperature, and maternal activity (49).

In the endocrine system, circadian rhythms control production of the glucocorticoid stress hormone cortisol. In humans, serum cortisol concentration peaks between 0600 and 1000 h and declines to its lowest point between 1800 and 0200 h (50). Plasma cortisol concentration is also controlled by feedback mechanisms of the hypothalamic-pituitary-adrenal (HPA) axis. During pregnancy, plasma cortisol concentration increases significantly, but circadian regulation of the timing of production is unchanged, suggesting that HPA-axis regulation is altered (51). The increase in maternal cortisol may play a role in fetal lung maturation and brain development (52) and help dampen maternal stress signals to protect the fetus. Support for this idea comes from a study in which non-pregnant women produced elevated cortisol after immersion in ice-cold water, but third-trimester pregnant women did not (53).

In addition to cortisol, two important hormones in human pregnancy are progesterone and estrogen, both of which are secreted in a circadian manner. During gestation, progesterone peaks during the dark hours, whereas estrogen concentration is lowest at night and peaks during the day (54, 55). During early gestation, progesterone and estrogen are synthesized and secreted by the ovaries. By mid-gestation, the placenta takes over production of these two hormones, and estrogen is also produced by the uterus. Both progesterone and estrogen, acting via their respective receptors, play vital roles in initiating and regulating uterine decidualization to allow for embryo attachment and placental development (56, 57). Estrogen promotes progesterone synthesis, helping to gradually increase progesterone throughout gestation (58). Progesterone's immunosuppressive properties (59) help prevent rejection of the fetus. Additionally, progesterone helps maintain uterine quiescence by interacting with progesterone receptor B (PR-B). Parturition is triggered when progesterone is functionally withdrawn, PR-B expression decreases, and progesterone receptor A (PR-A) expression increases. This switch causes an increase in procytokine release and initiation of myometrial contractions (60, 61). Later in this review, we further discuss the roles of melatonin in promoting parturition.

Metabolism, which is circadian regulated, is altered during pregnancy to meet the needs of the growing fetus. For example, glucose and fatty acid mobilization in the liver in the non-pregnant state are under circadian regulation. Given that the fetus depends on glucose and fatty acids, Wharfe et al. hypothesized that liver expression of clock genes might be altered during pregnancy. They tested this idea in mice and found that circadian rhythmicity of several clock genes was reduced during pregnancy. Additionally, expression of several glucoregulatory genes (62) and genes involved in liver metabolism were altered (63). For example, the genes encoding the lipolytic enzymes hormone–sensitive lipase and adipocyte triglyceride lipase showed clear rhythmic expression before pregnancy but lost rhythmicity at the onset of pregnancy. This metabolic adaptation is thought to allow continuous mobilization of fatty acids in response to fetal growth demands. Whether melatonin influences these changes beyond its roles in the circadian system is not yet known.

Core body temperature fluctuates in a circadian fashion in response to signals from the SCN. In humans, body temperature is highest around 1800 h and lowest around 0500 h, fluctuating by about 0.5°C around a 37.0°C median, or mesor. These changes in temperature, which must be small to permit activity of thousands of enzymes in the body (64), help entrain the peripheral oscillators (65). However, pregnancy appears to alter temperature regulation. In a study of 15 pregnant women, core body temperature measured at mid-day was highest during the first trimester (37.1°C) then decreased gradually throughout gestation, reaching its nadir (36.4°C) at 12 weeks postpartum, then increased and stabilized at 36.7°C by 24 weeks postpartum (66). A similar effect was observed in pregnant mice and rats. This lowered maternal body temperature is thought to facilitate heat transfer from the highly metabolically active placenta and fetus (49, 63, 67). The role of melatonin in regulating body temperature during pregnancy has not been investigated.

The final maternal adaptation to pregnancy addressed here is circadian regulation of activity. We found that, in mice, the daily time of onset of activity (monitored on a running wheel) shifted earlier at the beginning of pregnancy, then returned to the pre-pregnancy time by the end of gestation. Similarly, by using activity watches to measure women's daily activity, we found that the time of sleep onset shifted earlier during the first and second trimesters and then returned to the pre-pregnancy time during the third trimester. Additionally, we found that pregnant women had longer sleep duration and more activity during their inactive (sleep) phase than they did before or after pregnancy (68). Our results were consistent with other publications showing that pregnancy significantly reduced total daily activity in mice and humans (69, 70). For instance, Gamo et al. found that energy intake and body mass increased while body temperature and physical activity decreased in pregnant MF1 outbred mice (70). Additionally, Rousham et al. used accelerometers and self-reported interviews to show that human physical activity significantly decreased as pregnancy progressed from the second to third trimesters (69). How these circadian changes in maternal activity and sleep timing are controlled remains to be determined.

## Regulation and Functions of Melatonin During Pregnancy

**Figure 1:**
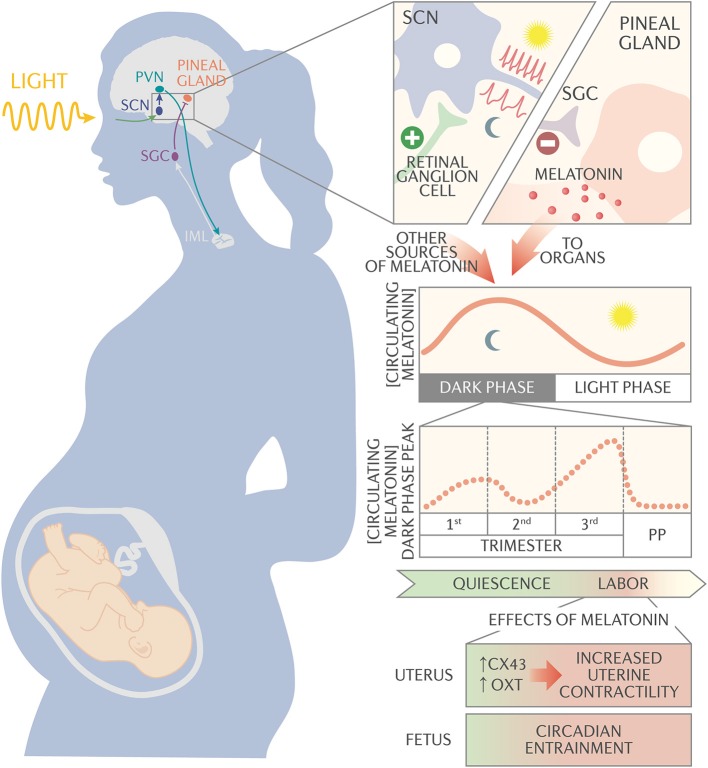
Schematic of melatonin circadian regulation and action during pregnancy. The suprachiasmatic nucleus (SCN) synchronizes to the external light/dark cycles via signals received from the melanopsin-containing retinal ganglion cells. The SCN neurons have a high firing rate during the light period and a slow firing rate during the dark period. The SCN projects to the paraventricular nucleus (PVN), which connects to the intermediolateral cell column (IML). The IML signals to the superior cervical ganglion (SCG), which signals to the pineal gland to synthesize and secrete melatonin into circulation. Melatonin, along with other neurotransmitters, synchronizes circadian rhythms throughout the body. During pregnancy, night time peak serum melatonin concentration decreases slightly between 1st and 2nd trimester, then begins to increase after 24 weeks of gestation until it reaches maximum concentration at the end of pregnancy. Serum melatonin acts synergistically with oxytocin via melatonin receptor on the uterus to activate membrane-bound phospholipase C and protein kinase C pathways. These pathways promote expression of the gap junction protein connexin-43 and increase uterine sensitivity to oxytocin, increasing uterine contractility. In addition, melatonin passes unaltered through the placenta and appears to be important for entraining fetal circadian rhythms.

Given the many ways in which physiology and circadian-regulated functions are altered by pregnancy, it is perhaps not surprising that melatonin secretion changes during pregnancy. In humans, the night-time peak serum melatonin concentration decreases slightly between the first and second trimesters, begins to increase after 24 weeks, reaches maximum by the end of pregnancy, and returns to the pre-pregnancy value by the second day post-partum (71) (see Figure 1). Although the precise mechanisms regulating the increase in melatonin are not fully known, one important factor may be the neuropeptide vasoactive intestinal polypeptide (VIP). Although VIP in the pineal gland likely controls melatonin synthesis (72), other VIP sources such as the fetus and placenta may be involved in regulating melatonin synthesis in pregnancy (72–74). To test the idea that placental-derived factors affect maternal melatonin production, Tamura et al. injected conditioned medium from cultured rat placenta into pregnant female rats, resulting in an increase in serum melatonin. The rat placenta cannot produce melatonin because it lacks the synthesizing enzymes, so the serum melatonin was produced by the mother (75), possibly in response to placental VIP. In humans, the placenta may directly contribute to increasing maternal plasma melatonin by synthesizing the hormone, which is amphiphilic and can thus cross into the maternal bloodstream. This mechanism may be especially important during the late third trimester, when peak melatonin is highest (71).

Just as placental melatonin can pass into the maternal bloodstream, maternal plasma melatonin can pass unaltered into the placenta. In the placenta, melatonin is thought to protect mononuclear villous cytotrophoblasts from apoptosis so they are able to continuously regenerate to fuse with and maintain a healthy syncytiotrophoblast layer (76, 77). This layer is in direct contact with maternal blood and mediates exchange of gases, nutrients, and wastes. One possible explanation for the initial decrease in serum melatonin in the first trimester is that placental mitochondria and polymorphonuclear leukocytes generate an abundance of superoxide free radicals (78, 79). The increase of ROS in the placenta may cause a temporary decline in maternal serum melatonin levels in order to protect the developing tissues from oxidative stress.

Failure to protect the placenta from oxidative stress can contribute to pregnancy complications. One common [2–8% of all pregnancies (80)] complication that involves oxidative stress is preeclampsia, which results in maternal high blood pressure and significant proteinuria. Preeclampsia arises due to a poorly developing placenta causing spiral arteries to form abnormally. This produces a decrease in the intervillous space volume and an increase in placental blood flow velocity, leading to placental hypoxia, oxidative stress, and mechanical injury (81). During preeclampsia, the concentrations of serum and placental melatonin are lower than in healthy pregnancies (71, 77). This may indicate that melatonin, acting as a free radical scavenger, is rapidly used up in the preeclamptic placenta. Given that melatonin can act as a strong antioxidant agent, is non-toxic, and can easily be administered, melatonin is being explored as an adjunct therapy in women with preeclampsia (82).

In addition to affecting the placenta, maternal melatonin appears to pass into the fetus (see Figure 1). This idea is supported by a study showing that changes in melatonin concentrations in umbilical cord artery and vein blood matched those in maternal plasma. Additionally, melatonin concentrations in maternal plasma and cord blood correlated significantly with administration of a single oral dose of melatonin to pregnant women undergoing cesarean section (83). In the fetus, maternal melatonin appears to be an important factor for entraining circadian rhythms. For instance, Torres-Farfan et al. suppressed maternal melatonin in capuchin monkeys and found that the timing of expression of the clock genes *BMAL-1* and *PER2* shifted in the fetal SCN. This shift was reversed when the authors delivered exogenous melatonin to the maternal circulation (84). Moreover, when the SCN was excised from hamsters, injection of melatonin into the mother was able to entrainment the pups (85). In another study, pregnant capuchin monkeys that were exposed to constant light had suppressed serum melatonin and an altered phase relationship between activity and temperature rhythm (86). Additionally, the newborns' temperature rhythms were desynchronized and had lower mesor. Maternal administration of supplemental melatonin restored synchrony and mesor to fetal temperature rhythms (86). In addition to helping entrain fetal circadian rhythms, melatonin likely also influences neurodevelopment and protects the fetus against oxidative stress. More detailed information regarding the effects of maternal melatonin on fetal rhythms is reviewed in other articles of this edition.

## Circadian Regulation of Parturition

Several lines of evidence reveal the importance of circadian rhythms in parturition. In most animals studied, parturition occurs just before or during the sleep/resting phase. For example, rats and hamsters, which are nocturnal, give birth during the daylight (87, 88). Mice, which are also nocturnal, give birth in the early morning just before the light period (89). In rats, complete ablation of the SCN changed the distribution of birth timing so that the animals delivered randomly throughout the day, including the dark phase (90). In addition to the SCN, peripheral clocks appear to participate in parturition timing, as mice in which *Bmal1* was knocked out in the uterine smooth muscle (myometrium) had 28% more deliveries during the daytime than the control mice, of which 92% delivered in the dark (91).

As in rodents, delivery is under circadian control in diurnal non-human primates, which tend to deliver during the early morning hours. For example, when rhesus monkeys were maintained in a normal photoperiod (lights off between 1900 and 0700 h), their uterine contractions reached maximum at 2300 h and they delivered at a mean time of 0115 h. To explore circadian regulation of parturition in baboons, Morgan et al. measured intraamniotic pressure and myometrial electrical activity. They found that contractures, which produced small intraamniotic pressure increases, occurred throughout pregnancy, but contractions, which caused larger intraamniotic pressure increases, always began at the onset of darkness (92).

In humans, delivery occurs at all hours but appears to be most common between 0200 and 0500 h (93, 94), and labor onset in both term and preterm birth most commonly occurs during the late night or early morning hours (between 2100 and 0600 h) (95–98) Additionally, after 24 weeks of gestation, uterine contractile activity develops a diurnal pattern with 67% of contractions occurring during the nighttime (99). This circadian regulation of delivery is likely controlled, in part, by melatonin (87, 100), as discussed next.

## The Role and Mechanism of Melatonin in Regulating Parturition

Late in human pregnancy, uterine contractions are strongest during the night, when peak melatonin concentrations are at their highest (71, 101), and the increase in peak melatonin at the end of pregnancy is thought to promote uterine contractions necessary for labor. Several animal studies supply evidence that melatonin has a strong influence on birth timing. For example, Takayama et al. reported that rats in which the pineal gland was removed, thereby eliminating melatonin production, delivered during the night instead of during the resting period of the day. However, when the authors injected the pinealectomized pregnant rats with melatonin at the beginning of the dark period, the rats delivered at a similar time as control rats (102).

Melatonin acts in concert with two other signaling molecules to promote contractions. First, it works with noradrenaline, as the α1 and α2-adrenergic receptors are expressed in the myometrium and contribute to uterine contractility (103). Early experiments on rat caudal artery indicated that melatonin alone could not initiate smooth muscle contractions but that noradrenaline-induced smooth muscle contractions were enhanced by melatonin (104, 105). Likewise, Martensson et al. reported that, in the presence of noradrenaline, melatonin induced potentiation of human myometrial contractions *in vitro* (106). Together, these data suggest that melatonin enhances noradrenaline-dependent contractions to help initiate labor.

Second, melatonin acts in cooperation with the non-apeptide oxytocin, which stimulates the myometrium by binding to G protein-coupled oxytocin receptors. The uterus becomes sensitive to oxytocin as the oxytocin receptors in the myometrium increase throughout pregnancy and reach their highest expression at the onset of labor (107). In *in vitro* experiments, telomerase-immortalized human myometrial smooth muscle cells treated with melatonin plus oxytocin were significantly more contractile than cells treated with oxytocin alone (108). However, in rats, which are nocturnal, melatonin appears to suppress rather than promote uterine contractions, as melatonin was shown to inhibit oxytocin-induced myometrial contractions *in vitro* (109). Additionally, it must be noted that some strains of laboratory mice, including C57BL/6, have a mutation in a key melatonin synthesis gene and thus do not produce melatonin but still deliver consistently in the early morning hours or just before dawn (110, 111). Thus, melatonin is not universally required to promote uterine contractions.

To enhance both noradrenaline- and oxytocin-stimulated contractions, melatonin appears to activate its receptor MTNR1B in the uterus. Evidence for this idea comes from the work of Sharkey et al., who treated immortalized human myometrial cells with melatonin and oxytocin. When they added an MTNR1B antagonist, myometrial contractility was reduced to the level observed in cells treated with oxytocin alone (108). Consistent with these *in vitro* data, MTNR1A and MTNR1B are expressed in myometrium from both non-pregnant and pregnant women (112, 113), but MTNR1B expression was higher in samples from pregnant women than non-pregnant women and was higher in term laboring myometrial samples than in term non-laboring myometrial samples (35, 108, 114, 115).

Once it is bound by melatonin, MTNR1B activates membrane-bound phospholipase-C (PLC) and protein kinase-C (PKC) (116). This is similar to the mechanism of action for oxytocin, which binds to oxytocin receptor and activates the PLC/PKC pathway (117). PKC activation leads to activation of myosin light chain kinase, resulting in myometrial contractions (118, 119). Additionally, melatonin appears to sensitize myometrial cells to oxytocin by leading to phosphorylation of caldesmon, which then releases its inhibition of actin-myosin cross-bridging and thus promotes even stronger uterine contractility (108, 120) (see Figure 1). In support of this model, treatment of human immortalized myometrial cells with the PLC inhibitor U73122 completely abolished contractility in response to oxytocin and melatonin treatment. Additionally, pretreatment with an MTNR1B antagonist abolished melatonin-induced increase in myosin light chain phosphorylation. Taken together, these data suggest that melatonin acts synergistically with oxytocin via MTNR1B to activate PLC, thus activating myosin light chain kinase and increasing sensitivity to oxytocin-mediated signals. Melatonin also activates PKC and increases expression of the gap junction protein connexin-43, resulting in increased myometrial coupling and stronger contractions (108, 120, 121).

## Circadian Disruption Can Affect Delivery

Given the importance of circadian rhythms in aligning physiological processes to environmental cues, it is not surprising that disruption of circadian rhythms (chronodisruption) has numerous negative health consequences (122) and may lead to preterm birth. Chronodisruption can occur in one of two ways. First, people can have mutations in the core clock genes, such as polymorphisms causing their sleep/wake cycle to be misaligned to the normal 24-h day (123). Second, people can experience environmental factors that force them to sleep or wake at times different than their body's biological clock. For example, 75% of people in the developed world use an alarm clock to wake up on workdays, causing “social jetlag” that is equivalent to traveling across multiple time zones (124). Additionally, many people have jobs that require them to shift their daily schedule by multiple hours during the week. Such shiftwork schedules can increase the risk for negative pregnancy outcomes including miscarriage, low birth weight, and preterm birth (125–127). For example, in one study of 845 pregnant women, night-time shift workers had higher rates of preterm birth [20 vs. 15%, adjusted odds ratio 2.0, 95% confidence interval (CI) 1.1 to 3.4] and low birth weight (9 vs. 6%, adjusted odds ratio 2.1, 95%CI 1.1 to 4.1) than women who worked daytime shifts (128). Similarly, the Pregnancy, Infection, Nutrition study, which analyzed data from 1,908 pregnant women, noted that women who worked night shifts during the first trimester had a 50% increased risk for preterm birth (relative risk 1.5, 95% confidence interval 1.0–2.0) (129). Furthermore, a report of the Nurses' Health study noted an association between night shift work and increased risk for early preterm birth (<32 weeks) (130). Moreover, a recent meta-analysis suggested that rotating shiftwork increased the risk for pre-term birth by 13% (odds ratio 1.13, 95% CI 1.00 to 1.28, *I*^2^ = 31%), and fixed night shift work increased the risk by 21% (odds ratio 1.21, 95% CI 1.03 to 1.42, *I*^2^ = 36%) (131). However, these results must be considered cautiously, as other meta-analyses have found no association between shift work and preterm birth (132, 133).

An important confounder in these studies is that women work many patterns of shift work (e.g., working nights or shifting the work schedule by a few or several hours every few days) (134). Additionally, studies have not accounted for other factors that can cause chronodisruption even in the “control” groups, such as using an alarm clock to wake up on work days (124) or using sleep aids to fall asleep and stimulants to stay awake at unusual or inappropriate times (135). Furthermore, nutrition, physical activity, and stress (134, 136) can all contribute to chronodisruption. More detailed analyses are needed to fully assess the effect of chronodisruption on risk of preterm birth.

Data from animal studies more strongly support the idea that chronodisruption has negative reproductive outcomes. For example, rhesus monkeys maintained on a light/dark cycle in which lights were off from 1900 to 0700 h delivered at a mean time of 0115 h. In contrast, those maintained on a shifted cycle with lights off from 0800 to 2000 h delivered between 1330 and 1715 h (101, 137). Several rodent studies provide strong evidence that disrupting circadian rhythms can negatively affect pregnancy outcomes. In one such study, female mice containing a mutation in the gene *Clock* had irregular estrous cycles, increased fetal reabsorption, and an increased number of dams that carried to full-term but failed to deliver (138). In another study, *Bmal1* mutant female mice had prolonged estrous cycles and low serum progesterone concentrations, leading to embryo implantation failure and infertility (139). A third set of studies used mice homozygous for a null mutation in the gene VPAC2 receptor (*Vipr2*^−/−^), which is important for photic entrainment of circadian rhythms in the SCN. *Vipr2*^−/−^ mice could not sustain normal circadian rhythms, and the females had abnormally long estrous cycles, impaired delivery, and poor pup survival (140–142). A fourth study modeled shiftwork or chronic jetlag by subjecting wild-type mice to repeated phase delays or phase advances of the light/dark cycle, revealing that such environmental disturbances significantly reduced the percentage of mice that carried their pregnancies to term (143). Finally, in a study in which wild-type pregnant mice were exposed to a short (22-h) or long (26-h) light/dark cycle, the fetal reabsorption rate increased, fetal development was delayed, and fetal weight was decreased (144). Together, these well-controlled studies clearly indicate that chronodisruption can impair mammalian reproductive outcomes.

## Conclusion and Potential for Circadian-Based Interventions

Given the potential ease of interventions, researchers have begun exploring options to alter circadian rhythms to control the timing of birth. For example, investigators found that treating chronodisrupted capuchin monkeys with supplemental melatonin partially restored normal maternal and temperature rhythms (86). Furthermore, Olcese et al. exposed pregnant women at term to full-spectrum light at night, causing their serum melatonin concentrations to decrease and their contractions to diminish. This raises the possibility of using light exposure to delay the onset of labor by dampening maternal nighttime melatonin secretion (100). Similarly, white/bright therapy light has been speculated to treat seasonal affective disorder and may be beneficial to overcome preterm deliveries by helping reset or instill stronger circadian rhythms (145). Future work in this area will hopefully reveal whether or not such light or melatonin strategies can be used to control the timing of birth in humans.

## Author Contributions

RM and SE conceived and wrote the review. EJ, JF, KB, and EH contributed to the writing and editing of the review.

### Conflict of Interest Statement

The authors declare that the research was conducted in the absence of any commercial or financial relationships that could be construed as a potential conflict of interest.
